# Supplementary material for: Data-driven strategies for optimal bicycle network growth
Source: R Soc Open Sci. 2020 Dec 16;7(12):201130. doi: 10.1098/rsos.201130 (PMC7813224; doi:10.1098/rsos.201130)
Supplement: Budapest bicycle network growth; Manhattan bicycle network growth [file rsos201130supp1.pdf]

Supplementary information

Data

Table SI.1 shows measures for the fifteen analyzed cities. For each layer in a city we report the number of nodes N and the number of connected components CC.

	walk			bike			rail			drive			Population
	N	CC	$\ell(km)$	N	CC	$\ell(km)$	N	CC	$\ell(km)$	N	CC	$\ell(km)$	
Amsterdam	23,321.0	1.0	2,075.67	34,529.0	355.0	972.08	1,096.0	8.0	288.72	15,125.0	1.0	2,010.49	872,680
Barcelona	20,203.0	1.0	2,122.6	7,553.0	122.0	229.19	263.0	29.0	105.98	10,393.0	1.0	1,551.44	1,600,000
Bogota	81,814.0	1.0	8,686.51	9,760.0	171.0	367.33	166.0	12.0	20.2	62,017.0	1.0	7,383.69	7,412,566
Budapest	73,172.0	1.0	7,746.12	10,494.0	257.0	336.13	1,588.0	20.0	522.06	37,012.0	1.0	5,332.97	1,752,286
Copenhagen	30,746.0	1.0	2,286.66	13,980.0	321.0	417.01	276.0	3.0	123.56	15,822.0	1.0	1,547.3	2,557,737
Detroit	47,828.0	1.0	6,769.46	3,663.0	53.0	141.06	20.0	3.0	11.54	28,462.0	1.0	5,624.49	672,662
Jakarta	140,042.0	1.0	13,947.96	248.0	19.0	8.44	60.0	8.0	81.24	138,388.0	1.0	14,194.2	10,075,310
LA	89,543.0	1.0	14,329.92	14,577.0	230.0	653.16	173.0	9.0	90.82	71,091.0	1.0	13,324.46	3,792,621
London	270,659.0	1.0	23,846.62	62,398.0	3,023.0	1,281.71	2,988.0	38.0	1,045.39	179,782.0	1.0	18,154.52	8,908,081
Manhattan	13,326.0	1.0	1,320.78	3,871.0	105.0	111.42	349.0	5.0	197.51	5,671.0	1.0	1,022.13	1,628,701
Mexico	108,033.0	1.0	14,547.18	5,218.0	52.0	332.37	371.0	18.0	253.48	95,375.0	1.0	13,732.39	8,918,653
Phoenix	111,363.0	1.0	14,314.0	35,631.0	141.0	1,221.18	105.0	4.0	71.64	73,688.0	1.0	11,841.49	1,445,632
Portland	50,878.0	1.0	5,324.78	24,252.0	198.0	596.36	230.0	2.0	132.36	35,025.0	1.0	4,583.47	583,776
Singapore	82,808.0	1.0	8,633.13	12,981.0	104.0	339.39	683.0	14.0	428.66	50,403.0	1.0	6,635.37	5,638,700

Table SI.1. Measures for the administrative area of analyzed cities. The number of connected components (CC) and nodes (N) for each layer in all cities of our dataset are highly diverse due to the varying developmental levels and focus of transport.

Figure SI.1 shows the connected component size distribution $P(N_{cc})$ for all considered layers and cities.

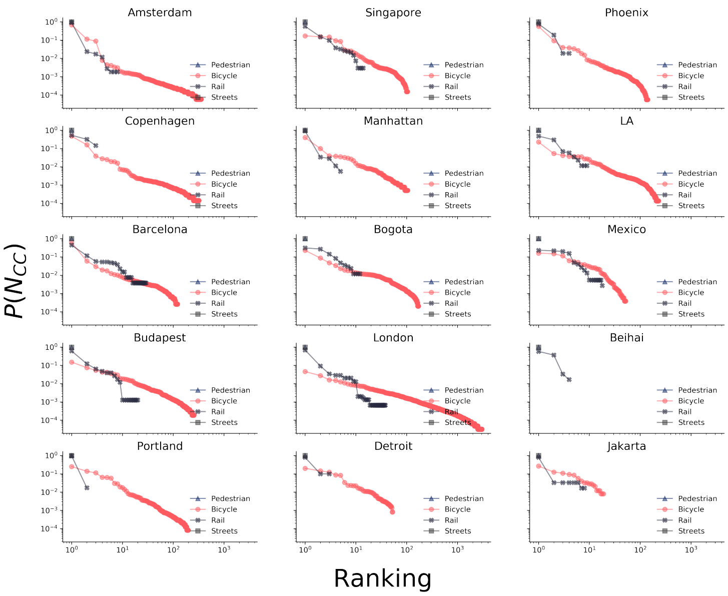

Figure SI.1. The connected component size distribution [$P(N_{cc})$] for all cities and layers is well connected except in the bicycle layer. London has the most fragmented bicycle infrastructure layer, with more than 3000 components

Algorithms

We develop two main algorithms to improve the bicycle layer. The first algorithm, *Largest-to-Second*, identifies in each step the largest connected component and connects it to the second largest. The second algorithm, *Largest-to-Closest*, also identifies the largest connected component, but in each step connects it to the closest of the remaining components.

To evaluate our algorithms we test them against a random baseline, *Random-to-Closest*. In each step, *Random-to-Closest* picks a component at random and connects it to the closest of the remaining components. We implement another baseline, the extreme case of *Closest-Components*, which prioritizes connecting the closest two components.

Algorithm 1 Largest-to-Second

procedure *L2S*
  $G \leftarrow$ bicycle network graph
  $wcc \leftarrow$ components of network G
loop for n-1 components in wcc:
  sort *wcc* by components size
  $cc \leftarrow$ two biggest components from *wcc*
  $i\_j \leftarrow$ closest nodes between cc_0 and cc_1
  connect cc_0 and cc_1
  goto *loop.*
close;

Algorithm 2 Largest-to-Closest

procedure *L2C*
  $G \leftarrow$ bicycle network graph
  $wcc \leftarrow$ components of network G
loop for n-1 components in wcc:
  sort *wcc* by components size
  $cc_0 \leftarrow$ biggest component from *wcc*
  $cc_n \leftarrow$ clossest component to cc_0
  $i\_j \leftarrow$ closest nodes between cc_0 and cc_n
  connect cc_0 and cc_n
  goto *loop.*
close;

Bicycle network improvement

Here we show the improvement of the bicycle network after the implementation of the algorithms. We measure the improvement with four different metrics. Two of them implement the notion of connectedness: i) Fraction of nodes inside the largest connected component compared to the total number of nodes in the bicycle layer, and ii) the fraction of link kilometers inside the largest connected component. In Figure SI.2 and SI.3 we show these two measures for fourteen different cities. We also

Algorithm 3 Random-to-Closest

procedure *R2C*
  $G \leftarrow$ bicycle network graph
  $wcc \leftarrow$ components of network G
loop for n-1 components in wcc:
  $cc_{ran} \leftarrow$ random component from wcc
  $cc_n \leftarrow$ clossest component to cc_{ran}
  $i\_j \leftarrow$ closest nodes between cc_{ran} and cc_n
  connect cc_{ran} and cc_n
  goto *loop*.
close;

Algorithm 4 Closest-Components

procedure *CC*
  $G \leftarrow$ bicycle network graph
  $wcc \leftarrow$ components of network G
loop for n-1 components in wcc:
  $\Delta_{min} \leftarrow$ clossest components in wcc
  $cc_0 \leftarrow$ first component for Δ_{min}
  $cc_1 \leftarrow$ second component for Δ_{min}
  $i\_j \leftarrow$ closest nodes between cc_0 and cc_1
  connect cc_0 and cc_1
  goto *loop*.
close;

quantify iii) bicycle-to-car directness to answer the question "how direct are the average routes of bicycles compared to cars?". Finally, in order to measure the cumulative efficiency of our algorithms, we define the metric: iv) G_{LCC} as the relative gain of bicycle path kilometers in the largest connected component. In Figures SI.5 and SI.6 we report these two measures for all algorithms and cities considered.

Bicycle network and 30 km/hr streets

We applied the algorithms to the bicycle infrastructure and all the bikeable streets, those with a speed limit of 30 km/hr or less (see Figures SI.8, SI.9, SI.10, and SI.11).

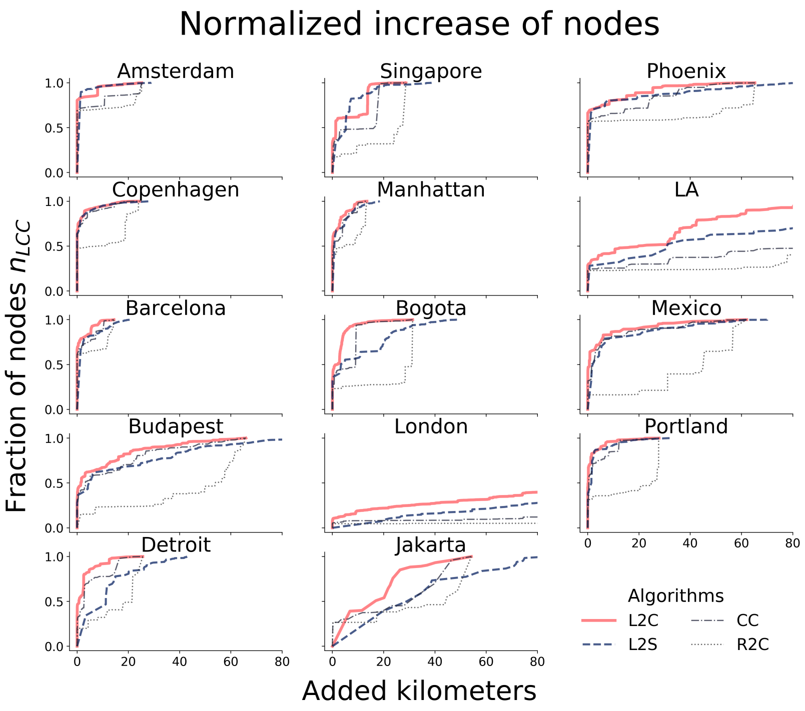

Figure SI.2. Normalized increase in nodes inside the largest connected component (n_{LCC}).

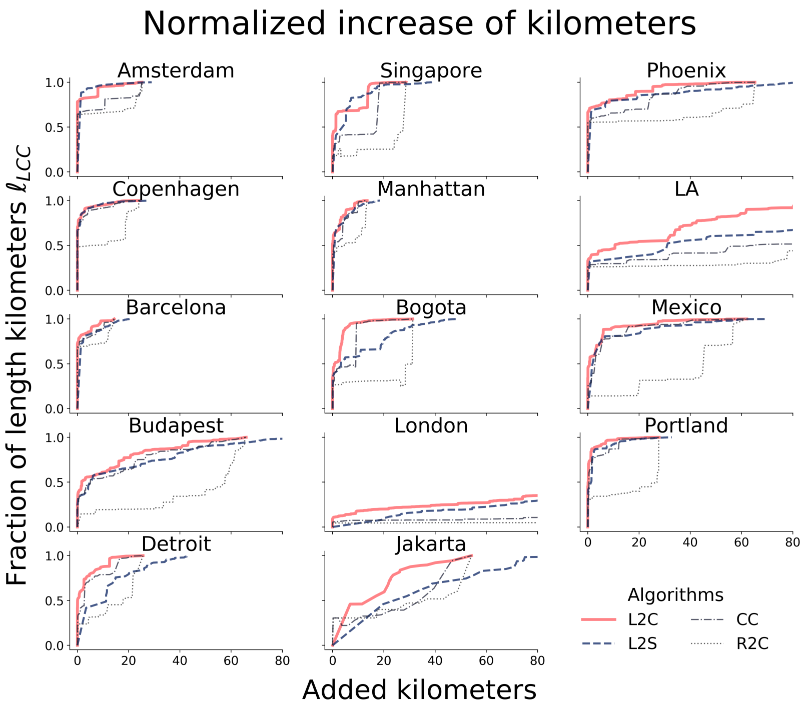

Figure SI.3. Normalized increase in kilometers inside the largest connected component (ℓ_{LCC}).

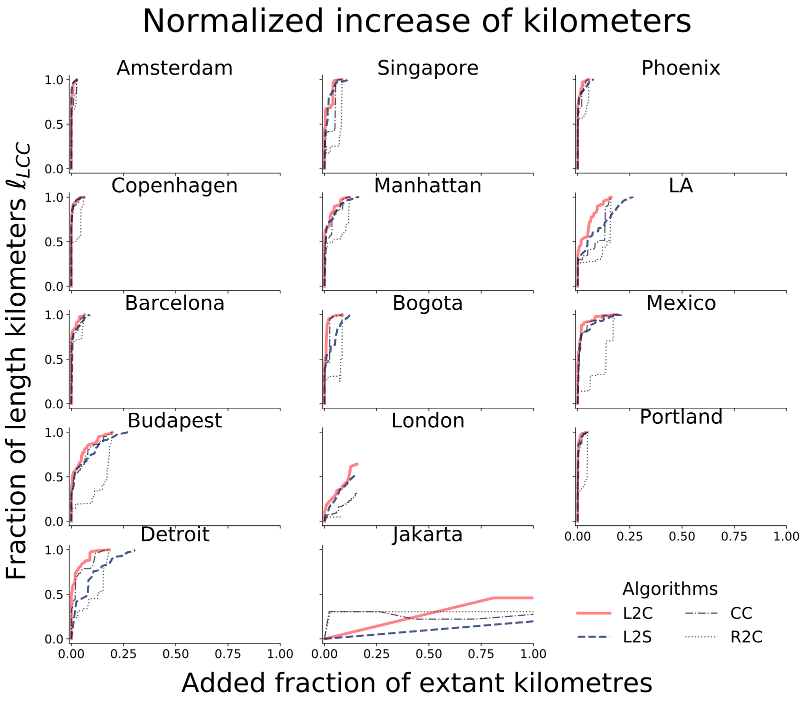

Figure SI.4. Normalized increase in kilometers inside the largest connected component (ℓ_{LCC}) versus the fraction of extant kilometers to be added.

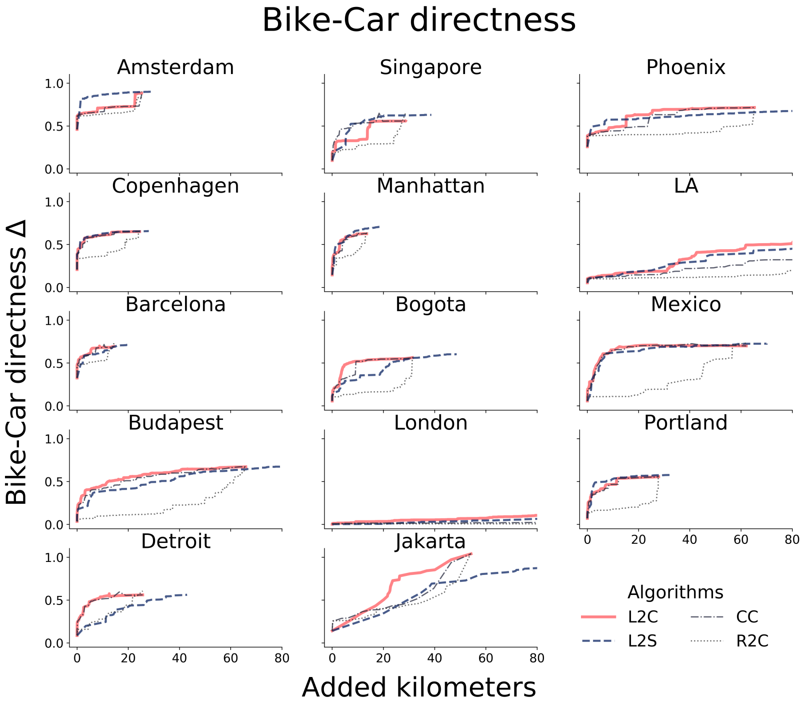

Figure SI.5. Bike-car directness Δ per invested kilometers.

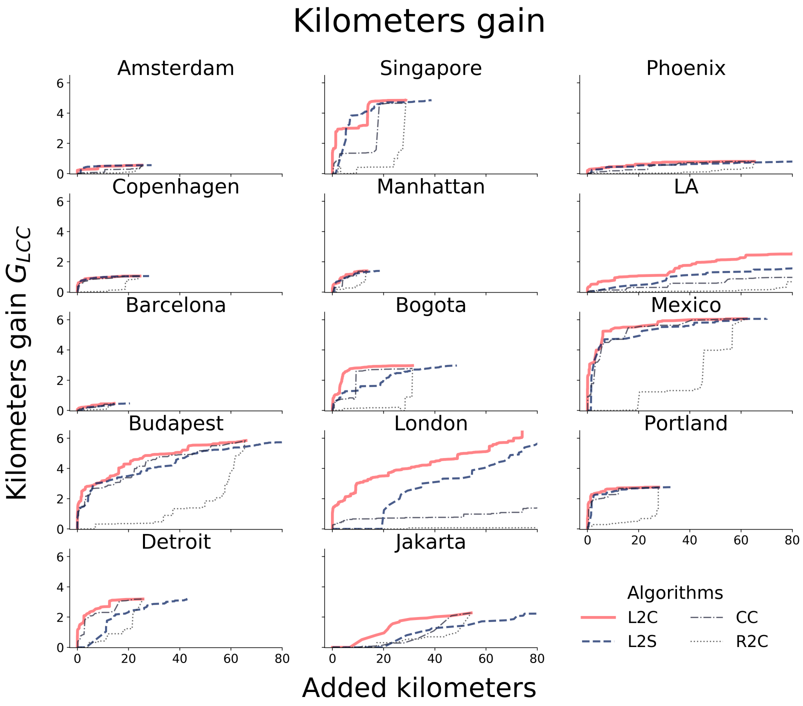

Figure SI.6. Kilometers gain in the largest connected component.

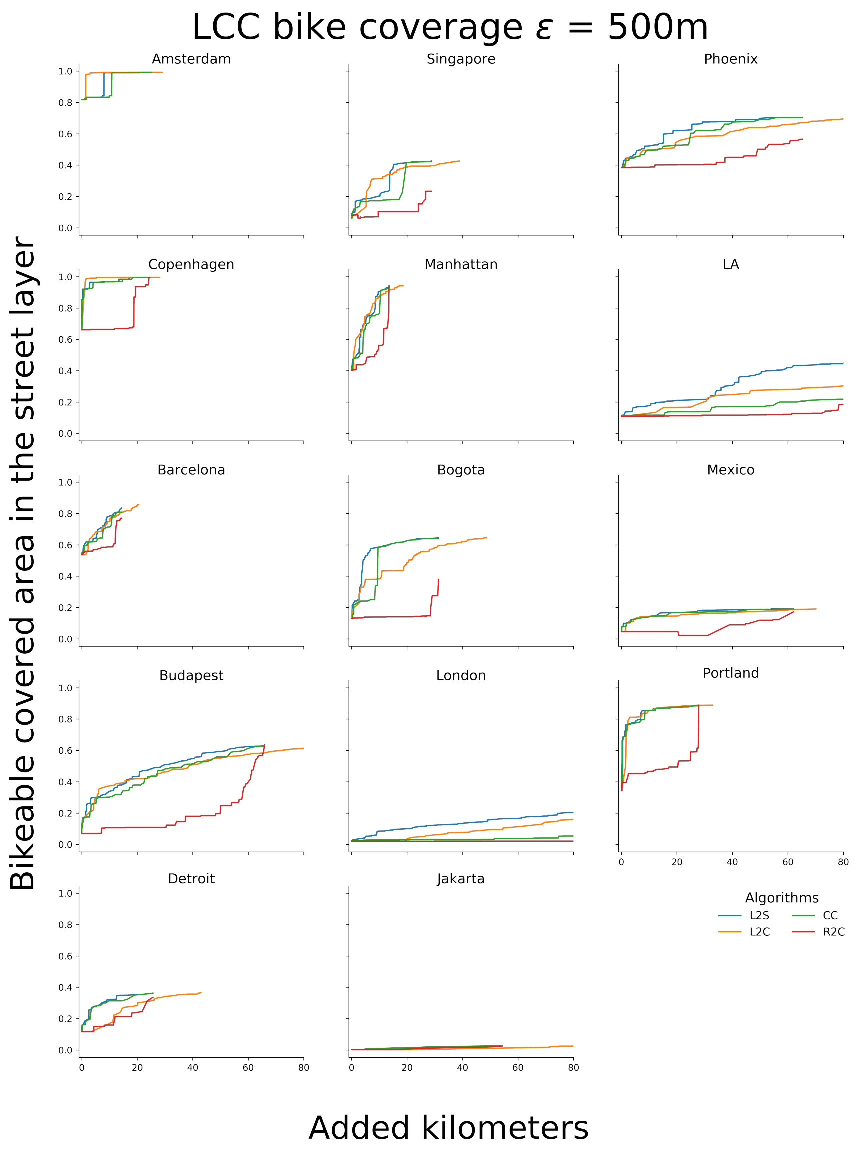

Figure SI.7. Area of influence (coverage) for the bicycle infrastructure in the street layer.

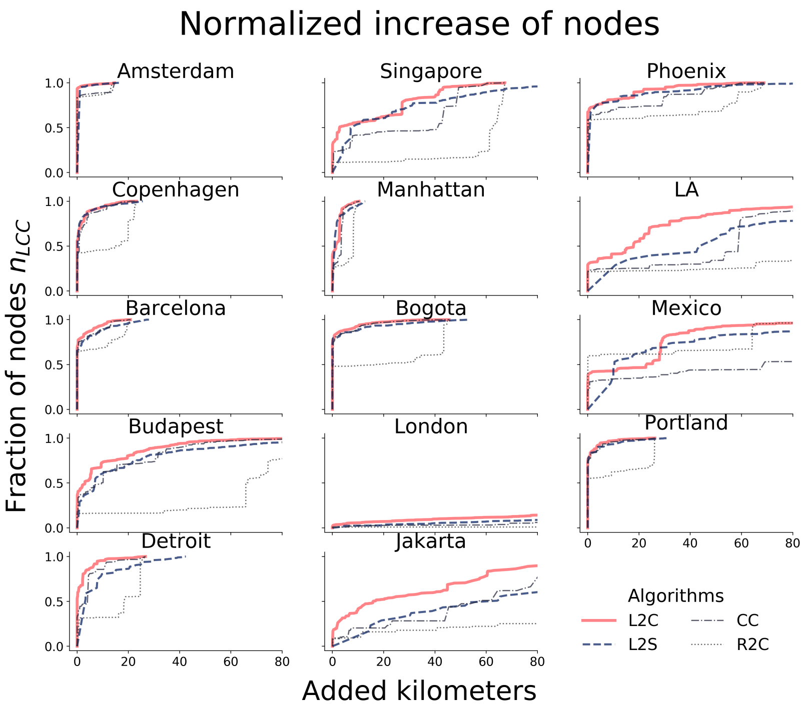

Figure SI.8. Normalized increase in nodes inside the largest connected component (n_{LCC}).

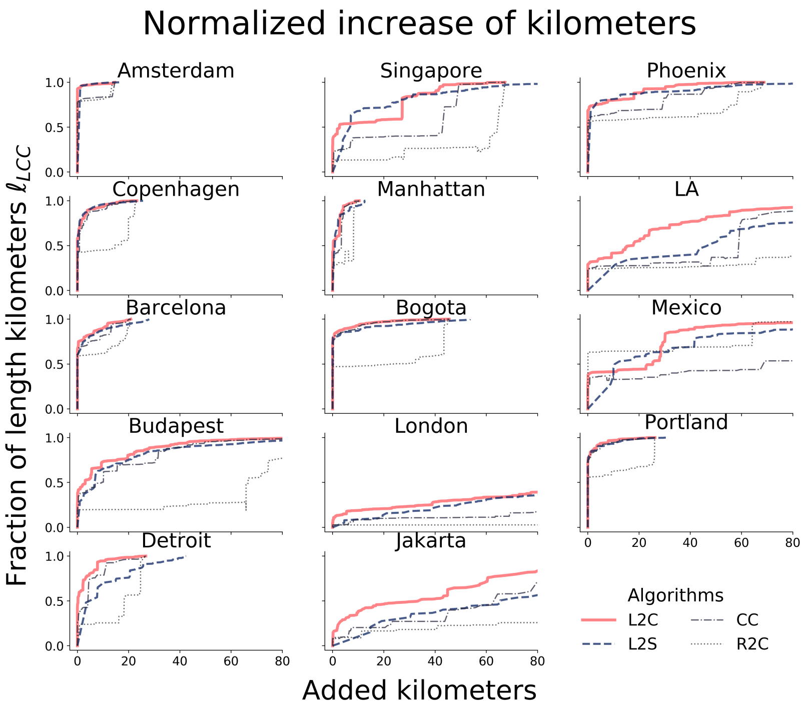

Figure SI.9. Normalized increase in kilometers inside the largest connected component (ℓ_{LCC}).

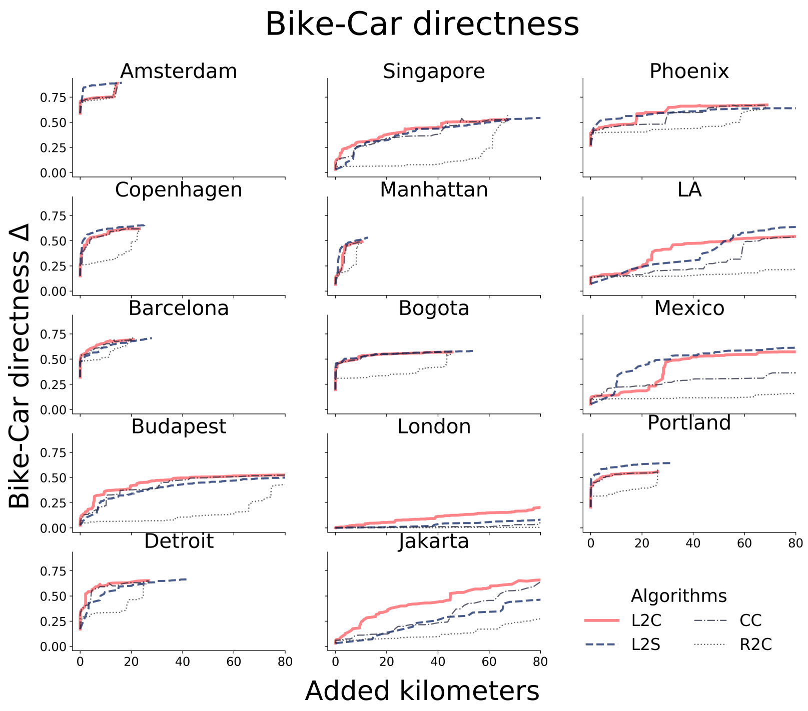

Figure SI.10. Bike-car directness Δ per invested kilometers.

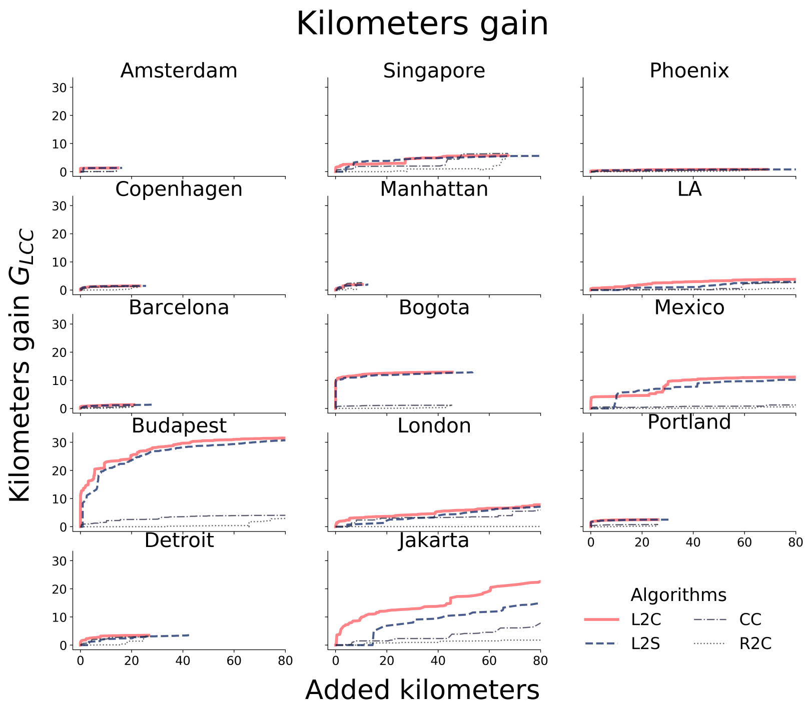

Figure SI.11. Kilometers gain in the largest connected component.
